# Impacts of physiological characteristics and human activities on the species distribution models of orchids taking the Hengduan Mountains as a case

**DOI:** 10.1002/ece3.10566

**Published:** 2023-10-01

**Authors:** Xue‐Man Wang, Pei‐Hao Peng, Mao‐Yang Bai, Wen‐Qian Bai, Shi‐Qi Zhang, Yu Feng, Juan Wang, Ying Tang

**Affiliations:** ^1^ College of Earth Sciences Chengdu University of Technology Chengdu China; ^2^ College of Tourism and Urban‐rural Planning Chengdu University of Technology Chengdu China

**Keywords:** biogeography, conservation, human activities, orchids, physiological characteristics, species distribution models (SDMs)

## Abstract

The biogeography research of orchids through species distribution models (SDMs), a vital tool in the biogeography field, is critical to understanding the fundamental geographic distribution patterns and identifying conservation priorities. The correspondence between species occurrence and environmental information is crucial to the model's performance. However, ecological preferences unique to different orchid species, such as their life forms, are often overlooked during the modeling process. This oversight can introduce bias and increase model uncertainty. Additionally, human activities, as an important potential predictor, have not been quantified in any orchid SDMs. Taking the Hengduan Mountains as an example, we preprocessed all orchid species' occurrences based on physiological characteristics. Choosing five spatial factors related to human activities to quantify the interference and enter into models as HI factor. Using different modeling methods (GLM, MaxEnt, and RF) and evaluation indices (AUC, TSS, and Kappa), diverse modeling strategies have been constructed in the study. A double‐ranking method has been adopted to select the critical orchid distribution regions. The results showed that classification models based on physiological characteristics significantly improved the model's accuracy while adding the HI factor had the same effect but the absence of enough significance. Suitability maps indicated that highly heterogeneous mountainous areas were vital for the distribution of orchids in the Hengduan Mountains. Different distribution patterns and critical regions existed between various orchid life forms geographically – terrestrial orchids were dominant in the mountain, and mycoherterophical orchids were primarily located in the north, more influenced by vegetation and temperature. Critical regions of epiphytic orchids were in the south due to a greater dependence on precipitation and temperature. These studies are informative for understanding the orchids' geographic distribution patterns in the Hengduan Mountains, promoting conservation and providing references for similar research beyond orchids.

## Graphical abstract

In this study, we considered physiological characteristics among orchids in orchid SDMs and quantified human activities as an environmental predictor input to the models. We validated the optimization of these two initiatives for orchid SDMs and suitability maps through various modeling strategies and validation approaches. We eventually combined the results of different models to filter out critical regions for the geographic distribution of orchids in the Hengduan Mountains.

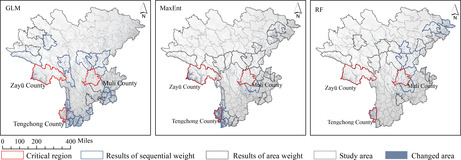

## 1 INTRODUCTION

Species distribution models (SDMs), as a capital method in biogeography research, are widely used in the response of target species under global climate change, the potential predicted distribution of invasive species, and the identification of conservation priorities (Araújo & Guisan, 2006; Austin & Van Niel, 2011; Bosso et al., 2022; Franklin, 2013; Guisan et al., 2013; Guo et al., 2020). Known as ecological niche models, it is a mathematical model established by species occurrence data and environmental information, estimating the ecological niche requirements of species based on statistical information provided by sampling sites and projecting to specific spatial and temporal regions to reflect the degree of habitat preference of species in a probabilistic form (Franklin, 2013; Guillera‐Arroita et al., 2015; Guisan & Thuiller, 2005; Guo et al., 2020; Naimi et al., 2014; Phillips et al., 2006). The model results reflect the suitable habitats in the geospatial, as a valuable reference and guidance for target species conservation.

The Orchidaceae is among the most species‐rich of all angiosperms family, widely distributed in all continents except Antarctica, reaching its highest diversity in the tropics, especially at middle elevations, and occupying a large part of local plant flora (Phillips et al., 2020). As actively evolving and rapidly diverging taxa, orchids feature prominently among lists of endangered plant species in some countries and regions, such as the South Americas, Australia, and China (Gaskett & Gallagher, 2018; Perez‐Escobar et al., 2017; Zhang et al., 2015), earning them recognition as flagship taxa for biodiversity conservation (Luo et al., 2003). Research showed that the diversity centers correspondence between orchids and other taxa (Anderson et al., 2008; Gaskett & Gallagher, 2018; Seaton et al., 2010). Thus, biogeographic studies of orchids provide a basis not only for their conservation efforts but also as a taxon specific enough to reveal the fundamental patterns of regional biogeography to understand the ecological relationships underpinning them and facilitate specific protective measures.

There are a few studies on the biogeographic distribution of orchids and their dependent ecological relationships utilizing SDMs. The correspondence between species locality and environmental information, the key to the model performance (Abrahms et al., 2019; Ancillotto et al., 2019; El‐Gabbas et al., 2021; McCune & Baraloto, 2016; Ranc et al., 2017), has been neglected in these studies (all occurrence data is treated as a whole and input into the model) (Crain & Fernandez, 2020; Djordjevic et al., 2016, 2020; Faruk et al., 2021; Tsiftsis & Tsiripidis, 2020; Wan et al., 2014). Plants have distinct functional traits that determine their environmental preferences (Schellenberger Costa et al., 2018), and this is equally true in orchids. Enough physiological and ecological research indicates distinct environmental requirements between various orchids, most significantly in different lifeforms (McCormick & Jacquemyn, 2014; Zhang et al., 2018). From a mathematic statistical point of view, the undistinguished occurrence data input may blur these differences and then increase the model uncertainty. Additionally, orchids have broad ecological fitness and are dependent more on the microenvironment (Kelly et al., 2013; Souza Rocha & Luiz Waechter, 2010), while biogeographic studies based on SDMs are usually conducted at a large spatial scale, such as a global biodiversity hotspot, signifying the target space is likely to contain enough heterogeneous environments to provide enormous ecological space for a variety of orchids. Ultimately, uncertainty in the correspondence may be transferred to spatial uncertainty in predicting suitability. Therefore, analyzing and validating the effects of physiological differences in orchid SDMs is critical to understanding orchid ecology, evaluating protected areas, and identifying conservation targets.

Species distribution patterns have developed under long‐term climatic stress and human disturbance (Wang, Yang, et al., 2023). Human activities and resulting habitat changes have caused and will continue to impact species distributions (Boivin et al., 2016). Lacking anthropogenic dispersal constraints and only using natural variables may lead to bias between species' actual and predicted distributions (Franklin, 2023). In orchid distribution pattern studies in Central America, model results show that most orchid hotspots occur in the most densely populated provinces (Crain & Fernandez, 2020). Although they can indicate the threat level to orchids outside protected areas, the absence of verification of ground‐truthing still does not rule out the possibility of prediction uncertainty in models only under natural predictors (Eyre et al., 2022). Human activity is generally recognized as one of the threats affecting orchids' geographic distribution, but there is no exact method to quantify or assess the impact of it (Anibaba et al., 2022; Crain & Fernandez, 2020; Djordjevic et al., 2020; Guisan & Thuiller, 2005; Hernandez et al., 2006; McCune & Baraloto, 2016). Some animal researchers have attempted to begin quantifying human activities and exploring their impact on the geographic distribution of target species (Mi et al., 2022; Qian et al., 2022; Zhang et al., 2022). Although these researches have generally positive results, employing only one modeling procedure makes it challenging to reveal the universality of human activities' effection on species distributions. Consequently, selecting appropriate indicators to quantify human activities and introduce them into orchid SDMs, together with validation and assessment using a multi‐modeling strategy, is significant for understanding the impact of human interference on the distribution of orchids and analyzing the current survival status for more efficient protection.

To further validate and confirm these issues, we used the case of the Hengduan Mountains. This region is one of the global biodiversity hotspots with a prominent representation of orchids among its flora. Different modeling approaches and validation methods were employed to explore the role of physiological characteristics and human activities in orchid SDMs. The following questions were addressed: 1. How do physiological characteristics and human activities impact orchid SDMs? 2. How do these factors affect orchid suitability prediction maps? 3. What are the orchid geographic distribution patterns and critical locations in the Hengduan Mountains based on different modeling strategies? These studies provide valuable insight into the geographic distribution patterns of orchids in the Hengduan Mountains and aid in assessing protected areas. Furthermore, the results can inform the modeling process for other species or regions.

## 2 MATERIALS AND METHODS

### 2.1 Data collection and preprocessing

The occurrence data of targeted species is related to the performance of SDMs. The public databases (such as GBIF, always obtain species occurrence from it) have proven to researchers that their data are not enough and existing sampling deviation (Beck et al., 2014; de Araujo et al., 2022; Garcia‐Rosello et al., 2023). Improving the accuracy of distributions cannot be ignored (Tulloch et al., 2016), as overlooking it could lead to significant conservation challenges or shortcomings. Thus, the quality of species occurrence data plays a more vital role than their quantity as long as meeting statistical requirements.

**FIGURE 1:**
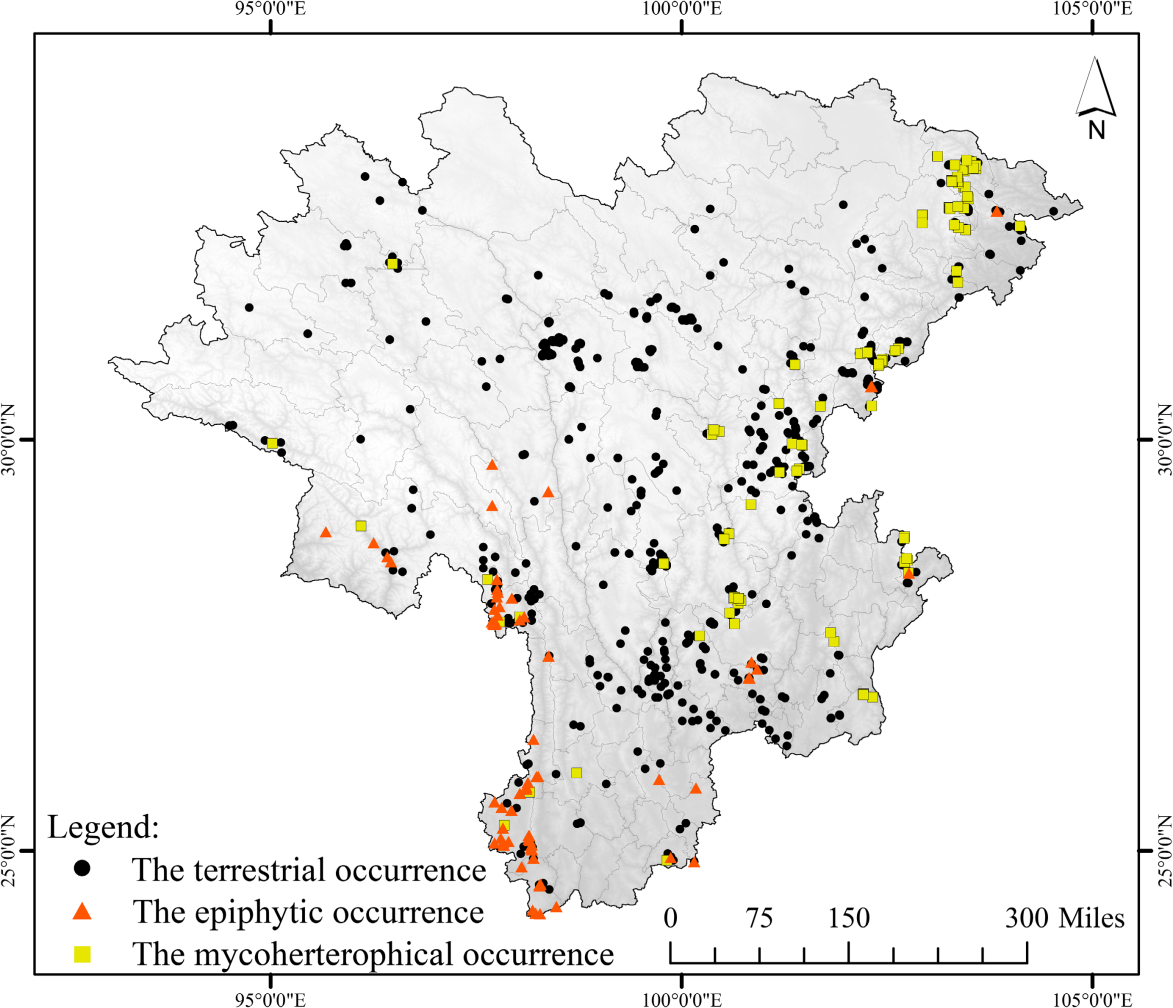
The location of the study area and the occurrence of different orchid lifeforms.

In this study, most orchid occurrence data were obtained from our field surveys in recent years (*n* = 10,470), which were recorded via a combination of transects and quadrats. The length of each transect was preset not less than 100 m; yet in practice, they were often more than 1 km. The vegetation type, topography, and geomorphological conditions were considered primarily when setting up these transects because a full investigation is hard to establish in a complex topographic area, and we tried to make the transects cover as much as possible of accessible habitats. We conducted an elaborate investigation within a range of 20 m on both sides, set up quadrats (5 × 5 m) where we found orchids and recorded these species and locations in detail. The interval of the quadrats was set to at least 10 m. Additionally, for the rare and endangered species recorded, the individual transects were set up in the possible habitats because they may have unique environmental preferences. Another small portion was obtained from the National Specimen Information Infrastructure (*n* = 963, http://nsii.org.cn/) that had been rigorously screened to ensure accuracy (accurate coordinates and species identification). Referring to Zhou et al.'s (2016) research on orchid lifeforms classification, we divided all orchid data into terrestrial (*n* = 10,794), epiphytic (*n* = 193) and mycoheterotrophic (*n* = 446). The details are shown in Figure 1 for the distributions of these occurrences.

### 2.2 Environmental variables selection

We accounted for biotic and abiotic variables that likely affect orchid communities and distributions, with four main categories in our models, bioclimatic variables, vegetation types, terrain attributes, and soil attributes, that have been proven the main components of orchid habitats and limiting factors for several orchid species physiologically and ecologically (Crain & Fernandez, 2020; Djordjevic et al., 2020; McCormick & Jacquemyn, 2014; Zhang et al., 2018). The latest 19 bioclimatic variables (30‐s resolution) were downloaded from the Worldclim database (Fick & Hijmans, 2017) (https://www.worldclim.org/), reduced variable collinearity with the Pearson correlation analysis, and remained five bioclimatic factors finally (|*r*| < .7, see detail in Appendix S1.1). Orchids distribute bound to particular vegetation and terrain features (i.e., elevation, slope, and aspect), each of which was included in our models due to potential effects on suitable temperature, water conditions, and stable pollinators for orchids survival (Acharya et al., 2011; Crain & Fernandez, 2020; Jacquemyn et al., 2005; Kelly et al., 2013). We used the 1:1 million vegetation map of China (Hou, 2019) from the Resource and Environment Science and Data Center (https://www.resdc.cn/) and the ASTER GDEM 30 M data from the Geospatial Data Cloud (https://www.gscloud.cn/) to generate the slope and aspect via GIS. Considering the geographical distribution preference of orchids influeced by the soil attributes and their mycorrhizal associates, four main soil attributes were included in our models (Figura et al., 2020; Kaur et al., 2021; Waud et al., 2016). This information was obtained from the National Science & Technology Infrastructure (http://data.tpdc.ac.cn/zh‐hans/) named the Soil map based Harmonized World Soil Database (v1.2). Detailed information about environment variables is available in Appendix S1.2.

### 2.3 Quantification of human activities

The human footprint could reflect human pressure comprehensively and objectively by selecting the spatial factors directly related to human activities (Buonincontri et al., 2023; Venter et al., 2016; Woolmer et al., 2008). Based on this, we put forward the HI factor, containing five indexes (population density, grazing density, human access, electrical power infrastructure, and land use/cover), regarded as a vital input variable in our models. The specific calculation and standardized method are as follows.

Ecological demand is always associated with population density (Liu et al., 2013). The Worldpop program (https://www.worldpop.org/) produces data on population distributions and characteristics at high spatial resolution. We downloaded the population density database and classified greater than 1000 people /km^2^ as 10 scores. For the rest, the scores 0–10 were calculated and assigned according to the logarithmic equation (Venter et al., 2016).

We collected the number of cattle and sheep (from the statistical yearbook: https://data.cnki.net/yearbook) and the areas in each county. We transformed (assuming one cattle's ecological consumption equals five sheep), referring to the literature (Yin et al., 2020), and concluded the grazing density based on the following formula:

$${\text{Grazing}}_{\mathrm{den}}=\frac{\log x_{i}}{\log x_{\max}}\times 10$$

where Grazing_den_ means the grazing density of each grid, $x_{i}$ represents the ratio of sheep number to the area in the county where the pixel is located, and $x_{\max}$ is the largest value in $x_{i}$.

Human access means that human activities could enter natural habitats through roads, which may reduce the environmental quality and the number of habitats (Geneletti, 2003). The distance from the road network (obtained from the National Catalog Service for Geographic Information: https://www.webmap.cn/) was a scoring criterion in our study (see Table 1).

**TABLE 1:** Human access scores criteria. Also quantified to between 0 and 10. As our region have no water transportation conditions, it was not considered.

Type	0–90 m	90–500 m	500–1000 m	1000–3000 m
Railway	8	6	4	2
National‐level highway	10	8	6	4
Provincial‐level highway	8	6	4	2
County‐level highway	6	4	2	0
Village‐level highway	4	2	1	0

Night light data represents the level of regional socio‐economic development and power infrastructure construction (Nordhaus & Chen, 2015), reflecting the ability and intensity of harness nature to some extent. A higher value means more frequent human activities. We scored the processed raster 1–10 by the quantile grading method after the preprocessing of the VIIRS Stray Light Corrected Nighttime Day dataset via Google Earth Engine (GEE).

Dissimilar land use has diverse effects on ecosystem change processes and the natural environment (Foley et al., 2005). According to the land use classification standards, we downloaded the database of the Globeland30 from the National Catalog Service for Geographic Information (https://www.webmap.cn/), assigning 10 points to construction land, followed by 7 points to arable land, 3 points to forests and irrigation, 1 point to grassland (taking into account the impact of grazing in mountainous areas), and 0 points to other land attributes (i.e., permanent snow and ice surface).

Finally, we summed all normalized layers with equal weights using the GIS raster calculator to obtain the HI layer. Combined with the above environmental variables, there were a total of 14 variables involved in our models (see detail in Appendix S1.2). All layers were resampled to 1 km and unified the same coordinate system.

### 2.4 Construction and evaluation of orchid SDMs

The filtering was applied to datasets classified by lifestyle. In detail, the spatial autocorrelation was limited to 1 km to avoid overfitting before running the model and eventually obtained four data sets for comparing physiology character effects in models (all‐data, t‐data, e‐data, and m‐data). Meanwhile, all environmental layers were resampled to 1 km and unified with the same coordinate system.

There are numerous algorithms for SDMs but no absolute superiority between them (Elith et al., 2006), which could be roughly categorized into mechanistic models, regression models, machine learning models, and ecological niche models (Bai et al., 2022). Considering algorithmic features and wide application (Norberg et al., 2019), we chose the generalized linear model (GLM), the random forest model (RF), and the MaxEnt models for exploring the effects of physiological characteristics and human activities in orchid SDMs. The construction of our models is based on the BIOMOD platform, a freeware and open‐source, is implemented in R, includes multiple algorithms technology, and can fit and compare different models (Thuiller et al., 2009, 2016). Even with default settings, achieve favorable prediction performance (Deka & Morshed, 2018; Uusitalo et al., 2019; Wang, Yin, et al., 2023; Zhao et al., 2021). Considering the available arithmetic resources, we set the interaction level of the GLM to 1 (default is 0) to characterize the interactions between the variables, the tree of the RF to 1000 (default is 500) to enhance the predictive power, and use default settings for the rest because of parameter tuning is not the focus of this study. To minimize the influence of sampling deviation, we randomly generated 2000 pseudo‐absence occurrences for every dataset (all‐data, t‐data, e‐data, and m‐data) and repeated them three times, thus we obtained three sets of data combinations containing presence and pseudo‐absence for each dataset. Each set of data was input into these algorithms for five repetitions. During each repetition, 70% of the dataset was randomly selected for training purposes, while the remaining 30% was used for validation. This process was repeated for each run. To distinguish clearly, we used the strategies combined with datasets to name each model. For example, G‐all represented an orchid distribution model built using GLM for all‐data. Additionally, for exploring the HI factor effect in models, we rerun all procedures under the condition without it. The full R code has been provided in the supplementary materials (Data S1).

We employed three indicators to evaluate the performance of our models: the area under the receiver operating character curve (AUC), the Kappa value, and the true skill statistic (TSS) (Ali et al., 2021; Li et al., 2023; Moameri et al., 2022). The AUC represents the probability, for a randomly selected observation, that the correct classification of the model is higher than the incorrect. Its value range is [0, 1], and the closer the value is to 1, the better the model will be. The Kappa coefficient means a ratio of the number of observation points correctly predicted to the incorrectly predicted. The TSS is an improved test index based on the Kappa coefficient. Both range from −1 to 1. When the value exceeds 0.4, the model has a bright prediction.

### 2.5 The critical regions of orchids' geographical distribution

The SDMs results of the species prediction are usually a continuous value of 0–1 to represent the probability of target species distribution. We set the threshold value as 0.5, thus more than it regarded as suitable for survival, otherwise as not having the conditions for species existence. All results were converted into binary data (1 represents existence; 0 represents nonexistence) and projected to the environmental layers via Arcmap GIS. For exploring the orchid lifeforms effect in models, we used spatial overlay technology, fused the suitability map generated based on t‐data, e‐data, and m‐data into a total layer, and compared it with the suitability map of all‐data.

Biodiversity conservation of target species chiefly commands the protection of their potentially suitable habitats. Hence, the amount of the suitability area within the specific geopolitically unit will become an essential consideration for conservation management to formulate policies and plans. To find out the critical regions of orchids distribution in the Hengduan Mountains, we separately counted their suitability habitat based on the county level. Instead of using a single area ranking to screen, which would confuse the various modeling strategies, we adopted a double‐ranking approach to compare the prediction suitability results between models. For each model, one of the screens was based on sequential weights, extracting the counties ranked in the top 30% according to the amount of area. Considering the existence of an enormous difference in the suitable area between counties, another was based on the area weights, we chose some counties at the top of the area sequence, and the sum of them was required to reach 50% of the total suitable habitat area in that column. The double‐ranking processing could exclude bias due to modeling differences that made it possible to compare the predicted results under arbitrarily modeled strategies.

## 3 RESULTS

### 3.1 Effects on the accuracy of orchid SDMs

The AUC values under all modeling strategies exceeded 0.8, with both Kappa and TSS values exceeding 0.4, indicating that our constructed models outperformed the random model and performed well in the prediction orchids habitat (see Appendix S2.1 for details of the model accuracy results).

**FIGURE 2:**
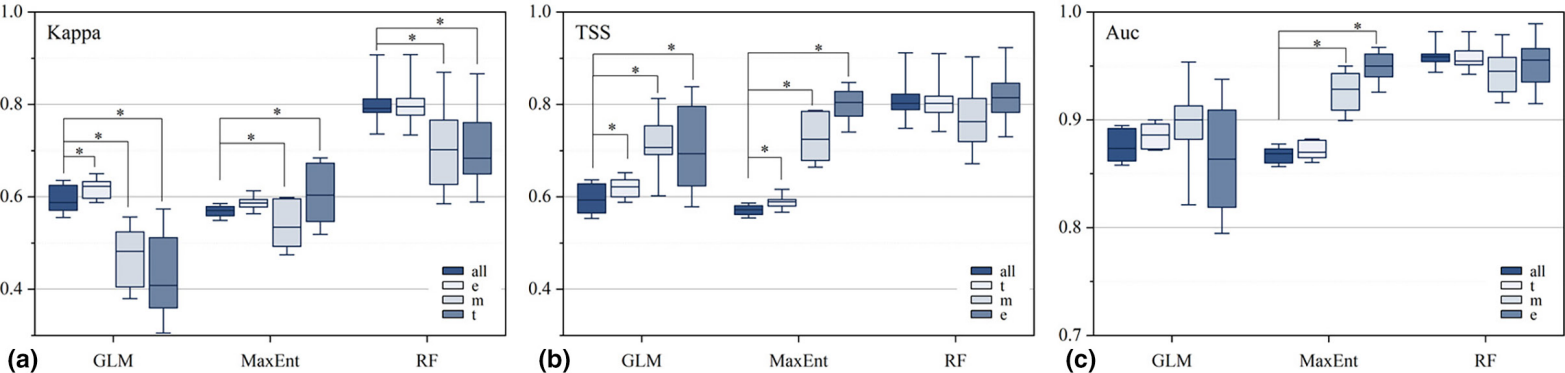
Accuracy of classification models versus all‐data models. The ALL represents modeling using overall orchids data. The e, m, and t represent the epiphytic, mycoheterotrophic, and terrestrial orchid models based on life‐type classification. The * represents the significance level of *p* <.05.

**FIGURE 3:**
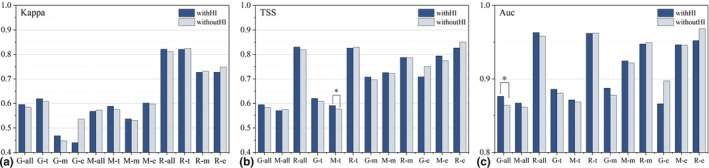
Comparison of model accuracy with and without HI factor. The G, M, and R represent the three modeling approaches of GLM, MaxEnt, and Random Forest. The a, t, m, and e represent the orchid dataset. Such as G‐a, meaning the species distribution modeled with all orchid data using GLM. The * represents the significance level of *p* <.05.

We compared the accuracy of the physiological classification models constructed based on physiological traits with the models generated by all‐data, obtaining 27 groups of comparative data under different approaches for modeling and verifying, of which 15 groups (55.6%) showed that the classification models had higher performance than the all‐data models. However, we also observed that only the R‐t model had the same pattern in the kind of RF models, while in the other comparison groups, the accuracy showed equal or slightly lower values (ranging from 0.001 to 0.09). When we temporarily discounted this kind, the proportional improvement of model accuracy by orchids lifeforms classification was able to reach more than 70%. We performed a *T*‐test on these differences and found general significant differences between physiological classification models and all‐data models (see Figure 2). The situation was quite distinct when we examined the effect of human influences on the accuracy. Based on the constructed models, we created 32 groups of comparative data, 23 of which showed (71.9%) that the accuracy of the orchid model with the inclusion of the HI factor was higher than that of the model without it, and in those comparisons, the maximum difference did not exceed 0.04. However, we also performed *T*‐test, and significance was present in only two groups (M‐ t and G‐all), which did not appear to be a generalization seemingly (see Figure 3).

### 3.2 Effects on the suitability maps of orchid SDMs

Ecological suitability maps of target species generated by SDMs are commonly an invaluable reference for biodiversity conservation planning. The effects of physiological characteristics and human activities on the accuracy of orchid SDMs are one of the objects of our exploration. However, more vital we wonder about their effects on the suitability maps. In our study, this variation was evident (see Appendix S2.2 for the detailed predicted suitability area by different modeling strategies).

**FIGURE 4:**
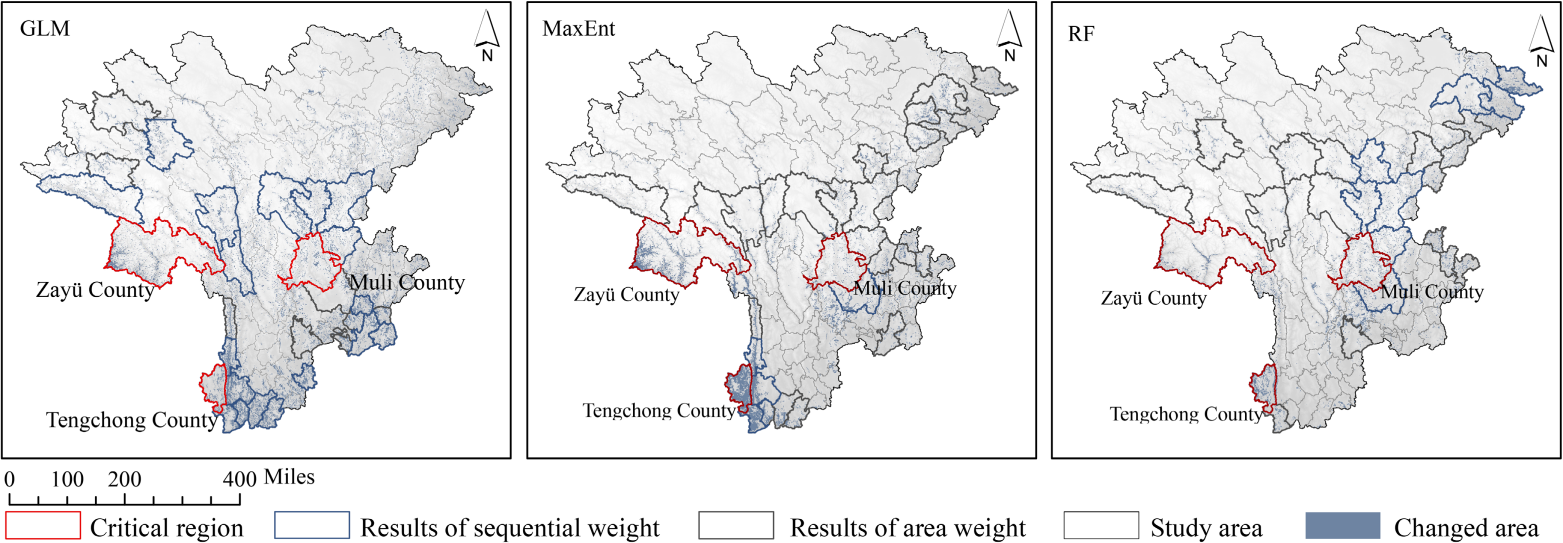
The changed area between the overall layer and the total layer; the red lines represent the results of the double‐ranking (see details in Appendix S3.1).

At first, we established the spatial comparison between the overall layer (modeled by all‐data) and the total layer (generated by superposition of the epiphytic, mycoheterotrophic, and terrestrial orchids suitability map), which showed that there was an enormous variance between them. In the GLM models, the changed area was 39,499 km^2^ (6.59% of the total study area). In the MaxEnt models, the changed area was 28,018 km^2^ (4.68%). In the RF models, the changed area was 19,974 km^2^ (3.33%). Moreover, it was usually shown that the predicted suitability area of overall layer was more than the total layer. To explore these discrepancy areas, we plotted the changed area map. Geopolitically, we counted the changed area for all counties separately in our study area. On this basis, we did the double‐ranking to find the critical regions in the change. The results showed that the eastern, western, and southeastern regions of the study area were the vital changed regions, especially Zayü County, Longyang District, and Tengchong City, which always was at the forefront of considering the sequential and area weights no matter the modeling strategies (see Figure 4).

**FIGURE 5:**
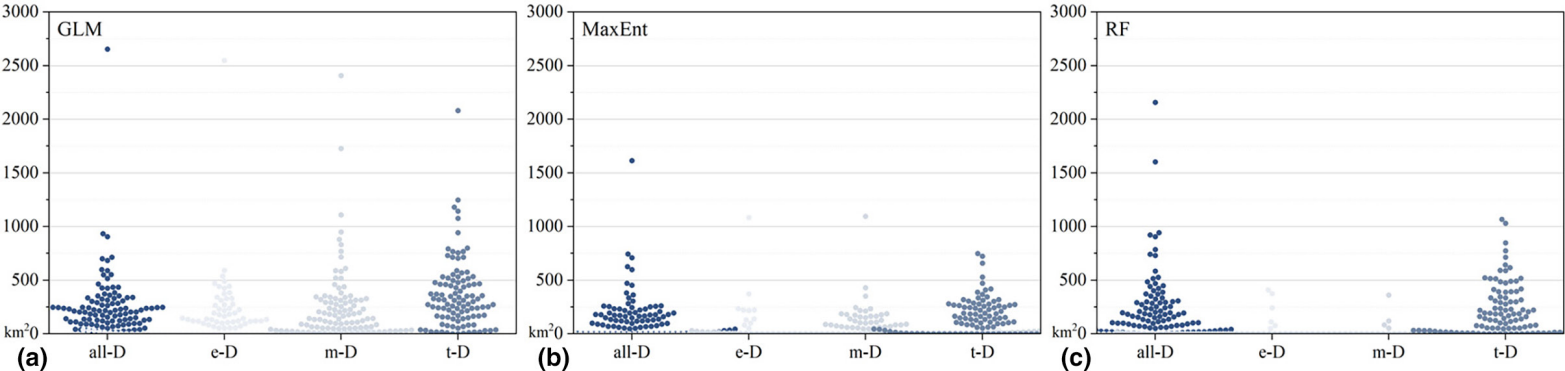
The *D*‐value in the predicted suitability area change with and without HI factors for all counties in the study area under different model strategies.

Second, in exploring the effect of human interference on suitability maps, we obtained 12 groups(see Appendix S2.2). The results showed that models containing the HI factor predicted fewer suitability areas than without this factor (more than 66%), with only four groups showing the opposite, namely, G‐m, M‐e, R‐e, and R‐m. To further explore the effect of the HI factor on different lifeforms orchids, we calculated the changed area in each county for all models separately and did the same double‐ranking exercise (see Appendix S3.2 for details) and plotted Figure 5. This figure indicated that when the HI factor was included in our models, the change in the predicted area caused by it had the greatest impact on terrestrial orchids, followed by mycoheterotrophic orchids and epiphytic orchids. Region analysis displayed that Zayü, Tengchong, and Yangyuan counties were the most changed in all comparisons (see Appendix S2.3, we have mapped the differences of all model strategies).

### 3.3 The orchid's geographical distribution pattern in the Hengduan Mountains

Mapping the suitability of orchids with all conditions (see Appendix S2.4), we intend to provide a reference for the conservation of orchids in the Hengduan Mountains. A wide range of suitable habitats for orchids existed in the Hengduan Mountains, both in the all‐layer and in the total layer, with the area of suitable habitats ranging from 58,510 km^2^ to 187,226 km^2^ (the differences caused by different models), with the lowest suitable area accounting for 9.77% (R‐total) and the highest reaching 31.25% (G‐all) of the total area of the study area. Suitability maps displayed noticeable geographic distribution centers. The primary distribution center was located in the northeastern part of the study area in the mountainous region with a north–south vertical distribution, while another center was located in the easternmountainous region. The distribution pattern observed in terrestrial orchids also exhibited a similar trend. The variable importance analysis supports this (refer to Appendix S3.4) as the ranking of terrestrial orchids remains consistent with the all‐data results, regardless of the modeling approach.

The terrestrial orchids distributed vary in area from 55,808 km^2^ (R‐t) to 160,956 km^2^ (G‐t), accounting for 9.31% and 26.86% of the total area. The situation was completely changed for mycoheterotrophic and epiphytic orchids; first, they were less widely spread than terrestrial orchids. The suitable area range of mycoheterotrophic orchids was 2742 km^2^ (R‐m) to 77,071 km^2^ (G‐m), accounting for 0.46% and 12.86% of the study area, respectively. The suitable area interval for epiphytic orchids was 3468 km^2^ (R‐e) to 30,637 km^2^ (G‐e), which accounted for 0.58% and 5.11% of the study area, respectively. Although the distribution pattern of mycoheterotrophic to that of terrestrial orchids, they tended to be found in the northeastern mountains (especially in M‐m and R‐m), while the distribution pattern of epiphytic orchids was more prominent, with the southern to the southwestern part of the study area being the preferrence. Similarly, in variable importance ranking, epiphytic and mycoheterotrophic orchids relied more on water (bio10 and bio13) and vegetation in partial physiological classification models.

**FIGURE 6:**
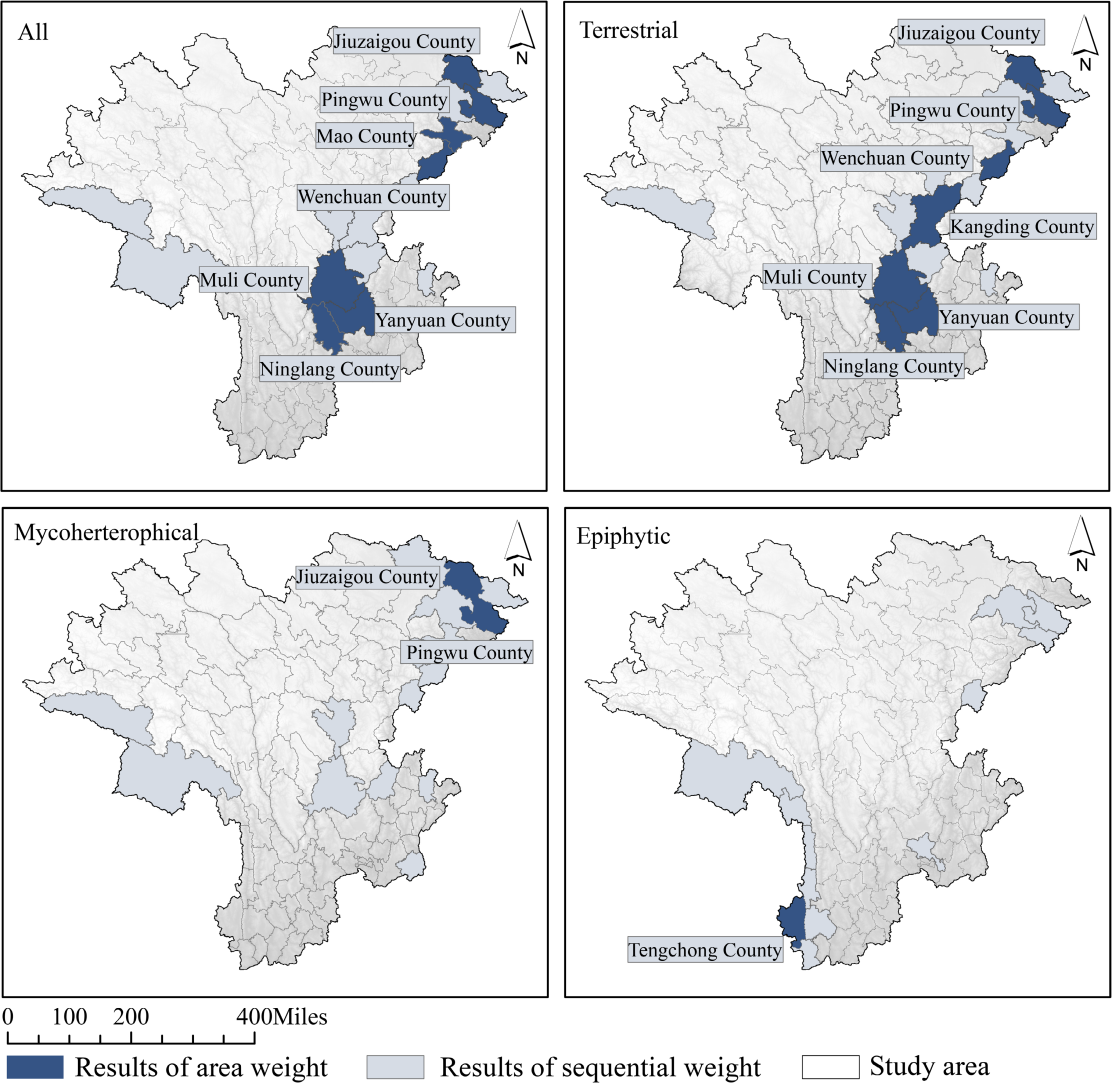
The suitability maps after double sorting of different data sets. The detailed process can be found in Appendix S3.3.

Using spatial analysis, we counted the suitable area of counties for all suitability maps and performed the same double‐ranking (see Appendix S3.3); this enables us to avoid the bias of prediction results caused by the different modeling approaches. The results indicated equally that the terrestrial orchids were almost consistent with the whole orchid ranking priority counties and area weighting counties, and the crucial areas were located in Muli, Yangyuan, Wenchuan, Jiuzhaigou, and Pingwu counties, and the sorting priority county for mycoheterotrophic orchids was the same as the first two, but the area weighting county was Jiuzhaigou County as far as the integrated model results were concerned. Epiphytic orchids also showed different results, and the suitable area ranking priority counties were located in the south to the southwest part of the study area, and the area weighting analysis result was Tengchong County (see Figure 6).

## 4 DISCUSSION

### 4.1 The importance of classification modeling based on physiological characters

Most orchids are in an actively evolving and specializing process from the biologically evolutionary aspect and are generally regarded as the flag group for biodiversity conservation (Luo et al., 2003). Their diversity hotspot was proven to correspond to other taxon distribution centers (Anderson et al., 2008; Gaskett & Gallagher, 2018; Seaton et al., 2010). Consequently, analysis of orchids' geographical distribution via SDMs makes it possible to understand regional fundamental geographical distribution patterns and identify priority conservation in a biodiversity hotspot (Crain & Fernandez, 2020; Souza Rocha & Luiz Waechter, 2010; Xing & Ree, 2017). SDMs are mathematical models established by the targeted species occurrences as well as environmental data that estimate the ecological niche requirements of species based on statistical information provided by sampling sites and mapped to specific spatial and temporal regions to reflect the degree of habitat preference of species in a probabilistic form (Araújo & Guisan, 2006; Dyderski et al., 2018; Elith & Leathwick, 2009; Guillera‐Arroita et al., 2015; Guisan & Thuiller, 2005; Guo et al., 2020; Ranc et al., 2017). The model results are the response to their suitable habitat distributions. However, the orchid family has shown their wide ecological suitability (Souza Rocha & Luiz Waechter, 2010) and significant physiological characteristics among different lifeforms (McCormick & Jacquemyn, 2014; Zhang et al., 2018). From the statistical point of view of SDMs, when we do not take measures to pretreatment of the orchid occurrences and directly input models, this would expand the environmental information provided by the sampling sites and may obtain an inaccurate and rough ecological requirement for orchids, thus affecting the model accuracy and suitability maps.

This has been confirmed in this study. Different modeling strategies and verification methods were adopted to test the physiological character effects on orchid SDMs. The result indicates that the models' accuracy would improve significantly when we confront and manage the physiological features, especially in epiphytic and mycoheterotrophic orchids. It is possible that the environmental relationship and dependence of these two types can be better represented by modeling separately. Another situation also proves the above conjecture that, without pretreatment for orchids, it may erroneously expand ecological niche requirements. In most of our model experiments, the predicted suitability area of unclassified tended to be higher than that results by the classification models.

Uncertainty in species distribution data is a factor that affects SDMs, which commonly includes uncertainty in the location of species occurrence, incomplete sampling, and selective bias (Guo et al., 2020). In this study, we put forward another situation that will cause the increase in model uncertainty: ignoring the pretreatment of targeted species occurrences data with inherent physiological differences. Not only limited to orchids, but the more precise matching of species occurrence with environmental information is also essential for species with distinct preferences, which is more common in dynamic SDMs studies of migratory animals in the ocean (El‐Gabbas et al., 2021). We emphasize that when serving the prediction of suitable habitats for target species using SDMs, in addition to optimizing the model structure, adjusting the model parameters, and improving the spatial resolution of the environment to improve the performance of models, it is necessary and efficient to pre‐process the data with the physiological differences embedded in the occurrence.

### 4.2 Impact of human activities on the geographical distribution pattern of orchids

Human actions are causing a biodiversity crisis (Brooks et al., 2006), which is a vital threat factor emphasized in most orchid conservation research (Acharya et al., 2011; McCormick & Jacquemyn, 2014; Zhang et al., 2015). Nevertheless, there are no exact approaches to evaluate the impact of human activities on the geographical distribution of orchids. Referring to the count of the human footprint index (Venter et al., 2016), we comprehensively quantify human activities, including grazing, roads, local economic level, ground cover, etc. However, the results indicated that the HI variable representing human interference did not perform the importance more than we thought regardless of model strategies. Although the accuracy of the models improves with the inclusion of this variable, it is not universally significant. In other words, human activities may have little impact on orchid distribution. Our investigated phenomenon precisely coincided with this view: a part of the orchid populations was found in fragmented forest patches close to towns.

What causes this phenomenon? Orchids generally occupy a relatively small ecological space and require more microenvironment (Djordjevic et al., 2020). As a result, they can survive on a small habitat patch, even if hardly. Such situations are not uncommon. In Costa Rica, several orchid hotspots still include some less primitive artificial environments, such as coffee plantations (Crain & Fernandez, 2020). Additionally, the orchids distribution pattern cannot be separated from the mycorrhizal environment dependent on the vegetation type (Selosse et al., 2004). Fortunately, with the improvement of people's awareness of ecological and biodiversity protection, the possibility of a specific habitat or vegetation completely disappearing due to human activities is reduced extremely. Physiologically, tiny, numerous, and long‐distance transmission capability seeds make orchids overcome some geographical obstacles, as we observed that most orchid populations show a highly dispersed spatial distribution pattern, which could resist disturbance caused by sudden environmental changes from human activities and affect little the geographical distribution pattern and the regional species pool. However, for some specific groups, the impact of human activities is crucial. Due to long‐term illegal collection, the wild individuality of *Gastrodia* R. Br., *Dendrobium* Sw., *Cymbidium* Sw., etc. has experienced a drastic reduction. Some endangered orchids with high environmental specificity distribute narrowly, and their endangerment mechanism is unclear. If the only habitat is inadvertently destroyed by human activities, it will lead to species extinction and loss of biodiversity.

However, the impact of interference is not all adverse. Djordjevic et al. (2020) suggests that some degree of disturbance has a positive effect on orchid performances by creating a space with favorable lighting conditions. The study by Jacquemyn et al. (2008) illustrates forest coppicing maintains viable populations of Orchis mascula in the long term. Considering pollinator diversity and reproductive success, Rewicz et al. (2017) proved roadside verges and edges of forests are better than those farther from the edges and roads exampling Epipactis helleborine. It is still worth noting that the distribution and germination of orchids are usually limited by the fungal distribution; at the same time, most orchidsflowers belong to deceptive pollination, and their pollination system is fixed and single (Givnish et al., 2015; Kelly et al., 2013; McCormick & Jacquemyn, 2014; Tremblay et al., 2005). Accordingly in the ecology niche, it is not easy to see orchids predominate in a certain habitat due to their competitiveness may be weaker than other species (Fekete et al., 2019). There is reason to believe that opportune interference may provide an ecological opportunity for orchids sometimes, which explains why most of the orchids are distributed in forest windows or beside the trails. From the perspective of the ecological landscape, environmental heterogeneity plays an important role in the diversity, differentiation, and rapid formation of orchid species (Perez‐Escobar et al., 2017) but does the landscape heterogeneity created unintentionally by human beings have the same effect? This needs further study.

Although human activities do not seem to produce much of a role in our model, we provide an available method for conservation managers in SDMs when they need to quantify human interference, with interchangeable choices of indicators for different purposes. For some specific regions or species, we cannot deny that human activities are probably the primary causes affecting their geographic distribution pattern.

### 4.3 Geographical distribution patterns of orchids in the Hengduan Mountains

Within global biodiversity hotspots, diversity and threats distribute unevenly (Cañadas et al., 2014; Harris et al., 2005; Murray‐Smith et al., 2009), and the harsh reality is that although funding for biodiversity is increasing, there is still a large gap between it and the actual resource needs (Waldron et al., 2013). Hence, understanding the geographic distribution patterns of species and identifying regional conservation priorities are more conducive to the use of special funding, optimizing the structure of funding, and improving the efficiency of conservation.

The Hengduan Mountains are one of the global biodiversity hotspots within which the orchid family is represented brilliantly (David, 2014; Marchese, 2015; Yu et al., 2020). Like other hotspots, the Hengduan Mountains have geographically diverse and highly heterogeneous environments to create favorable conditions for plant diversity, species formation, and dispersal (Wang et al., 2012). They are commonly considered important drivers of diversity (Crain & White, 2013; Perez‐Escobar et al., 2017). This may explain the breadth of orchid distribution in the Hengduan Mountains under either modeling strategy. According to the suitability maps, the terrestrial and overall orchids have a similar spatial distribution pattern. It is consistent with the research about the local orchid flora that the temperate terrestrial orchids occupy a critical component in the Hengduan Mountains (Lang, 1990). The results of the double‐ranking allow us to obtain some critical geographical regions of orchids, mainly concentrated in the Minshan Mountain System in the northeastern part of the study area, gradually extending southwestward to the Shaluri Mountain System (the widest range of the Hengduan Mountains) in the central part of the study area. These diverse mountainous areas could be consistent with the assertion of the peak level of diversity in the mountains (Acharya et al., 2011; Zizka & Antonelli, 2018). Other regional orchids' geographic research has demonstrated the rapid growth of mountain ranges and geological activities as the main drivers of orchid evolution and species formation (Crain & Fernandez, 2020; Dodson, 2003; Kirby, 2011).

The geographic distribution pattern of the mycoheterotrophic orchids largely coincided with the spatial patterns of the two above, but the double‐ranking results showed inconsistent critical regions. For mycoheterotrophic orchids, the middle and south ends of the Minshan Mountain Range (the easternmost mountain range of the Hengduan Mountains) in the northeastern part of the study area are the critical regions for their geographic distribution (Jiuzhaigou and Pingwu counties). This region belongs to a typical subtropical mountain climate with cold winters and cool summers, abundant rainfall but insufficient heat, and coniferous forests developed in high mountain valleys. Combined with the model variable importance results (see Appendix S3.4 for details), vegetation and bio10 (the hottest quarterly mean temperature) play a more significant role in the distribution model of mycoheterotrophic orchids. From this, we infer that the environmental preference of mycoheterotrophic orchids is for colder and wetter environments compared to terrestrial orchids in terms of temperature and precipitation. And may be more specialized to mycorrhizal environments generated by specific vegetation (Djordjevic et al., 2020; Kelly et al., 2013; Selosse et al., 2004). The geographic distribution pattern of epiphytic orchids is dissimilar. The southwestern mountains of the study area are the critical region for their distribution, which belongs to the Nujiang River valley and the Gaoligong Mountain system. The north–south longitudinal valley provides favorable conditions for the penetration of warm and humid airflow brought by the southwestern monsoon. The high temperature and abundant precipitation provide sufficient survival opportunities for epiphytic orchids. The analysis of environmental variables also showed that bioclimatic variables representing precipitation (bio13 and bio15) play a more significant role in the model of epiphytic orchids. This also coincides with the physiological characteristics of epiphytic orchids, where high temperatures and sufficient precipitation are the main ecological requirements (Zhang et al., 2018; Zotz & Hietz, 2001).

The environmental preferences of different life forms of orchids lead to geographically distinct spatial distribution patterns, suggesting that various geographic attributes could support separate centers of orchid diversity (Crain & Fernandez, 2020). It further illustrates that pre‐classification of orchid occurrences to achieve a more accurate match with environmental information enables distinction of these centers of diversity and better identification of conservation priorities.

### 4.4 Caveats and considerations for future research

In this study, we propose that the lack of preprocessing in orchid occurrences data brings uncertainty to the models. Classification by lifeforms increases accuracy in orchid SDMs. Although this is only a rough classification method based on physiological differences in orchids, we encourage researchers to attempt a further decomposition of it. It has been shown that distinct environmental preferences exist for different rooting systems of orchids (Stipkova et al., 2021). It would be a meaningful extension of this study to consider the physiological intervals of species as limiting thresholds to regulate environmental variables. For some endangered species, it is more significant for orchid biodiversity conservation to strengthen the correlation analysis with the environment, improve the resolution of environmental variables, and establish more accurate mathematical models. Another contribution of this study is to propose a method to quantify human activities in SDMs, which, although not shown to be important in our models, will be a reference for species conservation studies in other regions because the indicator we provide is replaceable. The final issue we would like to raise is the scale of applicability of the orchid SDMs. Research has shown that different spatial scales have a significant impact on the models' accuracy and suitability maps (Chen, 2023). What spatial scale for orchids could best optimize the performance is a worthy concern for future orchid conservation efforts. In addition, some results with different appearances in our model strategies may be influenced by the statistical algorithm or the sample size, which will be worthy of verification in a subsequent study. It could be seen that there are distinct differences between different model strategies in predicting suitability maps even with the same species occurrence dataset, environmental information, and resolution. Therefore ground‐truthing validation of SDMs would be a meaningful research direction.

## 5 CONCLUSION

Reducing the uncertainty of SDMs and improving the accuracy of model precision and predicted suitability map is among the unavoidable issues of SDMs applied to biogeography research. The extensive ecological adaptability of orchids may lead to more uncertainty in the correspondence between orchid occurrence data and environmental information when the absence of preprocessing occurrence data in orchid SDMs. This has been confirmed in our study. Multiple modeling approaches and validation demonstrate that classification modeling based on physiological characters enhances the accuracy of orchid SDMs. Such a feature is also reflected in the suitability maps of the model predictions. Mountainous areas with heterogeneous environments in the Hengduan Mountains hold the hotspots of orchids. But different living forms of orchids are influenced by distinct environmental variables, thus presenting diverse critical regions geographically. In addition, we propose a method for quantifying human activity, which could make it serve as a significant non‐natural variable in SDMs. It does improve model accuracy in our study but not a critical variable affecting the geographic distribution pattern of orchids. Importantly, we provide a method that can be borrowed and referenced by biogeographic studies of other regions or species, which is flexible and replacement. Our study emphasizes that model parameter tuning is an effective means to improve model performance. But for orchids, the classification modeling based on physiological characters and inclusion of human activities are equally essential for the positive effects. Ultimately, we expect that our study will inspire the thinking of related researchers in modeling SDMs to promote further biogeographic and conservation studies of orchids or other species.

## AUTHOR CONTRIBUTIONS

**Xue‐Man Wang:** Conceptualization (lead); data curation (lead); formal analysis (lead); investigation (lead); methodology (lead); software (equal); validation (equal); visualization (lead); writing – original draft (lead); writing – review and editing (lead). **Pei‐Hao Peng:** Conceptualization (supporting); funding acquisition (lead); methodology (supporting); project administration (lead); resources (lead); supervision (lead); writing – original draft (supporting); writing – review and editing (supporting). **Mao‐Yang Bai:** Data curation (supporting); formal analysis (supporting); investigation (equal); methodology (supporting); software (equal); writing – original draft (equal). **Wen‐Qian Bai:** Methodology (supporting); software (supporting); writing – original draft (supporting). **Shi‐Qi Zhang:** Data curation (supporting); investigation (supporting); methodology (supporting); software (supporting); writing – original draft (supporting). **Yu Feng:** Data curation (supporting); investigation (supporting); methodology (supporting); writing – original draft (supporting). **Juan Wang:** Funding acquisition (supporting); resources (supporting); writing – original draft (supporting). **Ying Tang:** Conceptualization (equal); data curation (supporting); formal analysis (supporting); investigation (lead); methodology (supporting); project administration (supporting); writing – original draft (equal); writing – review and editing (equal).

## CONFLICT OF INTEREST STATEMENT

The authors have no relevant financial or non‐financial interests to disclose.

## Supporting information


Data S1

Appendix S1–S2

Appendix S3

## Data Availability

Data supporting this study are available from Dryad (https://doi.org/10.5061/dryad.7d7wm380s). You can obtain it from this temporary link: https://datadryad.org/stash/share/0w6gY0SAbnAD5xlxpzpSZenSdJMZN‐6FzR2jNrTuoYQ. As some orchids are really rare and endangered, for protection, we upload the species occurrences after spatial autocorrelation calculation and replace their name with their lifeforms.
