# Supplementary material for: Impacts of physiological characteristics and human activities on the species distribution models of orchids taking the Hengduan Mountains as a case
Source: Ecol Evol. 2023 Oct 1;13(10):e10566. doi: 10.1002/ece3.10566 (PMC10542477; doi:10.1002/ece3.10566)

Appendix S1.1 The flowing figure drawn by RStudio showed the correlation of bioclimatic factors via the Pearson Correlation Analysis. The threshold adopted was |0.7|.


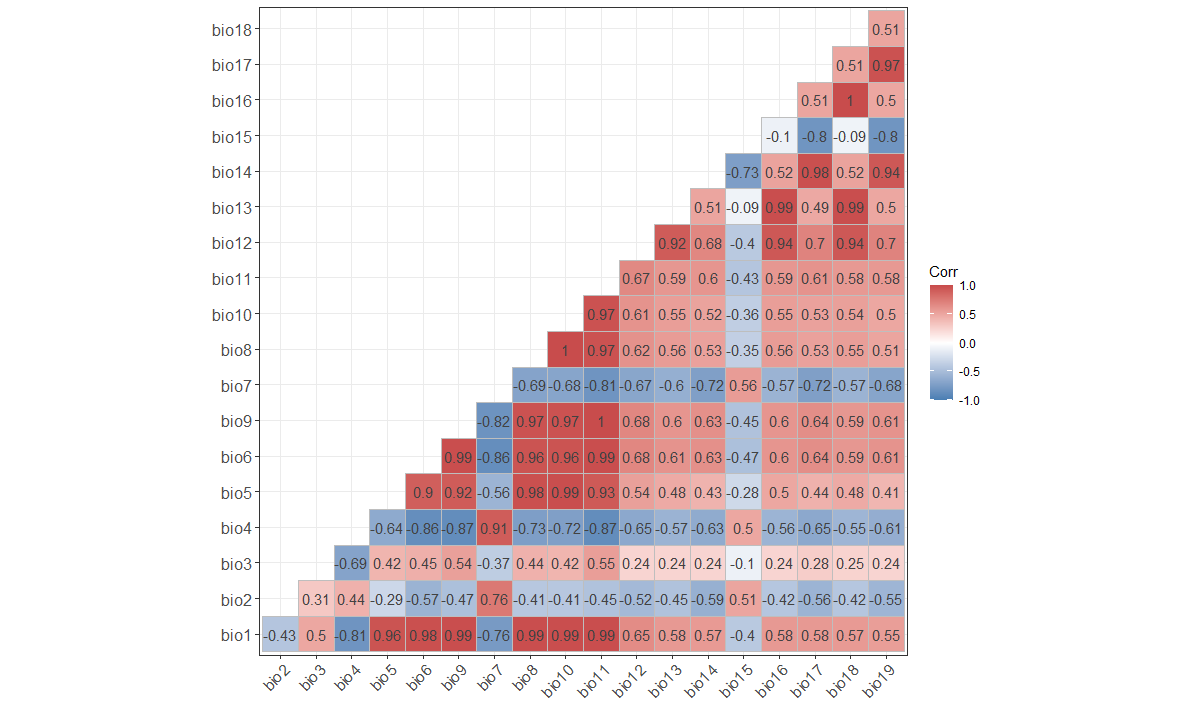


Appendix S1.2 The flowing table includes Environmental variables and their explanations. The variables selected for the potential distribution modeling of orchids are marked with an asterisk (*)..

| Variables | Explanations | Data source |
| --- | --- | --- |
| bio1 | Annual mean temperature | the WorldClim 2.1 database  (https://www.worldclim.org/) |
| bio2 * | Mean Diurnal Range |  |
| bio3 * | Isothermality ((bio2/bio7) × 100) |  |
| bio4 | Temperature seasonality (STD × 100) |  |
| bio5 | Max temperature of warmest month |  |
| bio6 | Min temperature of coldest month |  |
| bio7 | Temperature annual range (bio5–bio6) |  |
| bio8 | Mean temperature of wettest quarter |  |
| bio9 | Mean temperature of driest quarter |  |
| bio10 * | Mean temperature of warmest quarter |  |
| bio11 | Mean temperature of coldest quarter |  |
| bio12 | Annual precipitation |  |
| bio13 * | Precipitation of wettest month |  |
| bio14 | Precipitation of driest month |  |
| bio15 * | Precipitation Seasonality (Coefficient of Variation) |  |
| bio16 | Precipitation of wettest quarter |  |
| bio17 | Precipitation of driest quarter |  |
| bio18 | Precipitation of warmest quarter |  |
| bio19 | Precipitation of coldest quarter |  |
| vegetation * | / | 1:1 million vegetation map of China  from the Resource and Environment Science and Data Center(https://www.resdc.cn/) |
| elevation * | / | ASTER GDEM 30M data from the Geospatial Data Cloud（https://www.gscloud.cn/) |
| slope * | / |  |
| aspect * | / |  |
| T_gravel * | Topsoil gravel content | Soil map based Harmonized World Soil Database (v1.2) from the National Science & Technology Infrastructure (http://data.tpdc.ac.cn/zh-hans/) |
| T_sand * | Topsoil sand fraction |  |
| T_silt * | Topsoil silt fraction |  |
| T_clay * | Topsoil clay fraction |  |
| HI * | Human interference | Calculations in the article |

Appendix S2.1 the results of the model‘s accuracy. The “mean” means the average of the model operation, the “sd” means standard deviation, and “*” means that compared to all-data, the classification dataset model exists a difference on the 0.05 significance level.

| HFI factor | Test types | Kappa | | TSS | | AUC | |
| --- | --- | --- | --- | --- | --- | --- | --- |
|  | Models | mean | sd | mean | sd | mean | sd |
| contain HFI | GLM all | 0.596 | 0.038 | 0.595 | 0.039 | 0.876 | 0.017 |
|  | GLM t | 0.619^*^ | 0.029 | 0.620^*^ | 0.030 | 0.886 | 0.013 |
|  | GLM m | 0.468^*^ | 0.084 | 0.708^*^ | 0.099 | 0.887 | 0.062 |
|  | GLM e | 0.440^*^ | 0.126 | 0.708^*^ | 0.123 | 0.866 | 0.067 |
|  | MaxEnt all | 0.567 | 0.017 | 0.571 | 0.015 | 0.867 | 0.010 |
|  | MaxEnt t | 0.588^*^ | 0.023 | 0.591^*^ | 0.023 | 0.871 | 0.010 |
|  | MaxEnt m | 0.537^*^ | 0.058 | 0.726^*^ | 0.058 | 0.925^*^ | 0.024 |
|  | MaxEnt e | 0.601 | 0.078 | 0.794^*^ | 0.051 | 0.946^*^ | 0.020 |
|  | RF all | 0.821 | 0.081 | 0.830 | 0.077 | 0.963 | 0.018 |
|  | RF t | 0.821 | 0.082 | 0.826 | 0.080 | 0.962 | 0.019 |
|  | RF m | 0.727^*^ | 0.134 | 0.788 | 0.109 | 0.947 | 0.030 |
|  | RF e | 0.728^*^ | 0.131 | 0.827 | 0.091 | 0.952 | 0.035 |
| without HFI | GLM all | 0.584 | 0.021 | 0.583 | 0.022 | 0.864 | 0.013 |
|  | GLM t | 0.608^*^ | 0.020 | 0.608^*^ | 0.020 | 0.880^*^ | 0.009 |
|  | GLM m | 0.447^*^ | 0.103 | 0.696^*^ | 0.101 | 0.878 | 0.069 |
|  | GLM e | 0.536 | 0.099 | 0.750^*^ | 0.120 | 0.897^*^ | 0.063 |
|  | MaxEnt all | 0.573 | 0.013 | 0.575 | 0.012 | 0.861 | 0.008 |
|  | MaxEnt t | 0.575 | 0.018 | 0.577 | 0.017 | 0.869^*^ | 0.007 |
|  | MaxEnt m | 0.530^*^ | 0.059 | 0.722^*^ | 0.047 | 0.922^*^ | 0.023 |
|  | MaxEnt e | 0.598^*^ | 0.077 | 0.774^*^ | 0.056 | 0.946^*^ | 0.019 |
|  | RF all | 0.811 | 0.086 | 0.819 | 0.082 | 0.958 | 0.020 |
|  | RF t | 0.824 | 0.078 | 0.828 | 0.076 | 0.962 | 0.018 |
|  | RF m | 0.731 | 0.128 | 0.787 | 0.107 | 0.949 | 0.029 |
|  | RF e | 0.748 | 0.116 | 0.851 | 0.078 | 0.968 | 0.018 |

Appendix S2.2 The following table shows the suitability habitat area of different models. The D-value means the difference between the predicted suitability habitat area (with HF factor) and the suitable area (without HF factor). The CA means the changed area with or without the suitability habitats results of the HF factor. Its ratio represents the proportion in the total study area. The unit used is km^2^.

| Model types | With HF | Without HF | D-value | CA | CA ratio |
| --- | --- | --- | --- | --- | --- |
| G-all | 175552 | 178699 | -3147 | 27060 | 4.52% |
| G-t | 151415 | 157508 | -6093 | 37891 | 6.32% |
| G-m | 72384 | 66772 | 5612 | 25387 | 4.24% |
| G-e | 28164 | 29755 | -1591 | 13606 | 2.27% |
| G-total | 173883 | 182224 | -8341 | 56818 | 9.48% |
| M-all | 88312 | 91156 | -2844 | 15320 | 2.56% |
| M-t | 78912 | 82764 | -3852 | 15649 | 2.61% |
| M-m | 19619 | 19736 | -117 | 6535 | 1.09% |
| M-e | 13761 | 13581 | 180 | 3149 | 0.53% |
| M-total | 95268 | 98012 | -2744 | 27606 | 4.61% |
| R-all | 62755 | 66785 | -4030 | 24299 | 4.06% |
| R-t | 52760 | 61763 | -9003 | 21815 | 3.64% |
| R-m | 2673 | 2496 | 177 | 715 | 0.12% |
| R-e | 3171 | 2875 | 296 | 1278 | 0.21% |
| R-total | 55243 | 63531 | -8288 | 28737 | 4.80% |

Appendix S2.3 The following figure depicts the difference in prediction results of different model strategies with or without the HI factor.


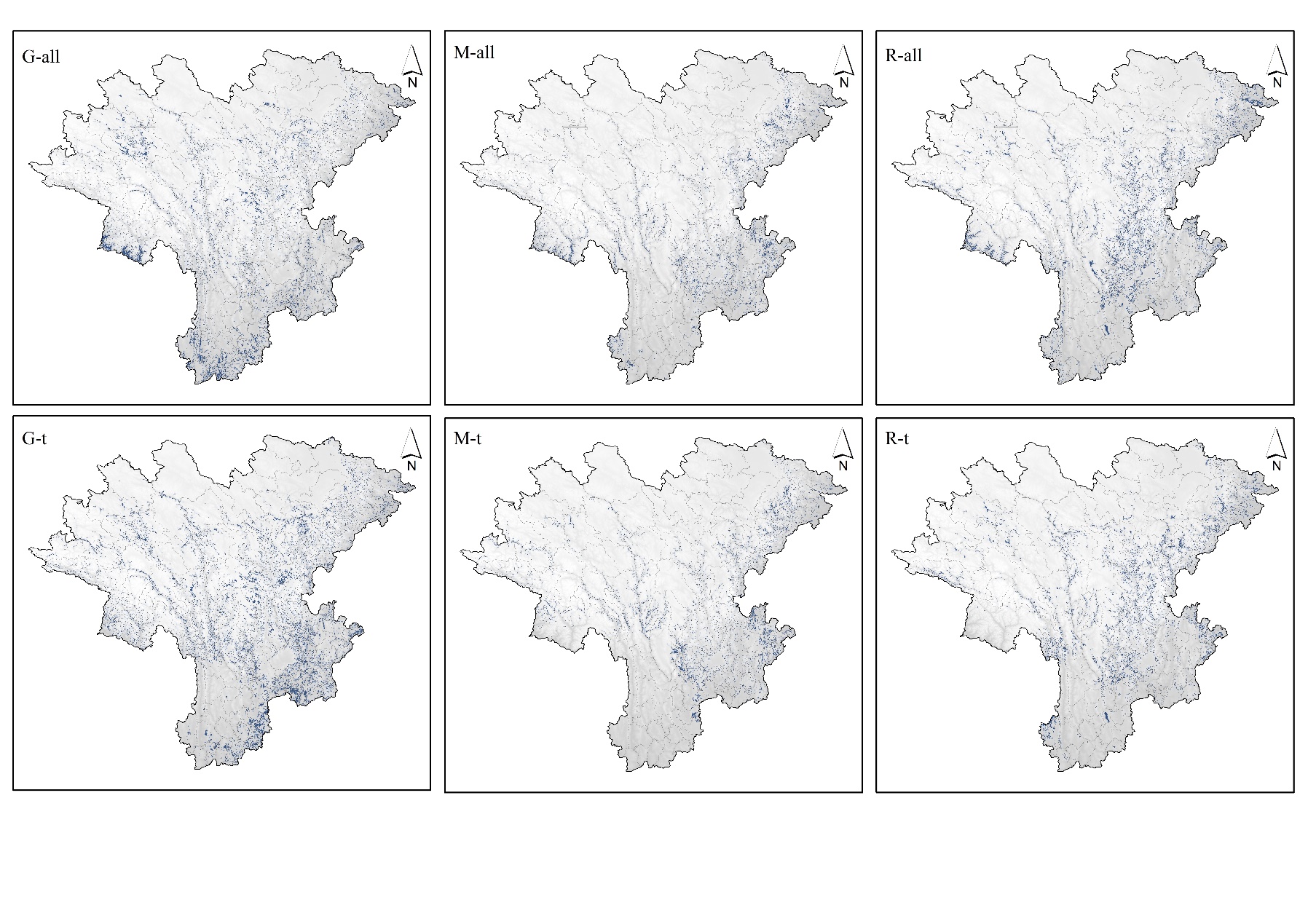


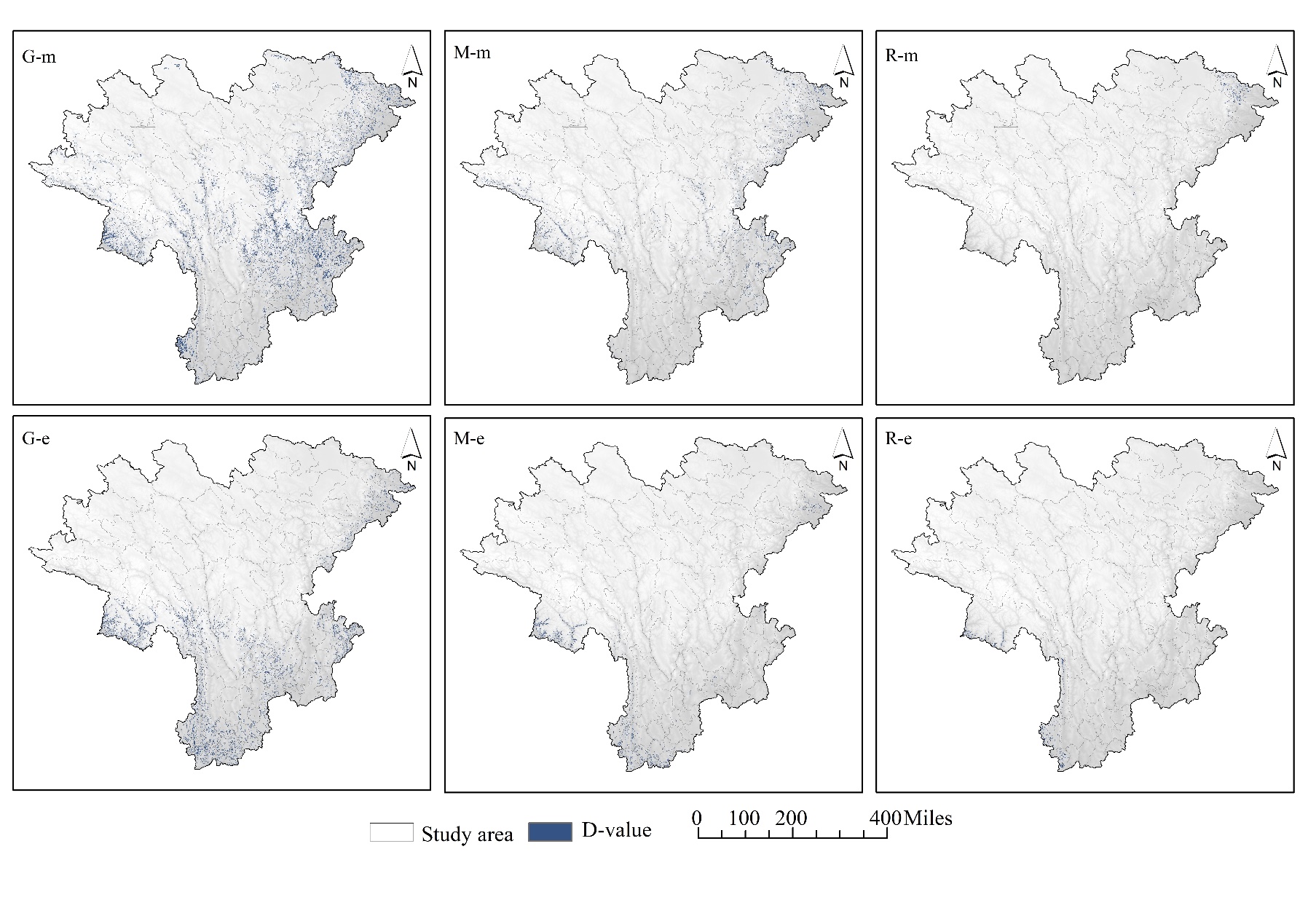


Appendix S2.4 The following figure depicts suitable habitat maps for orchids under different model strategies


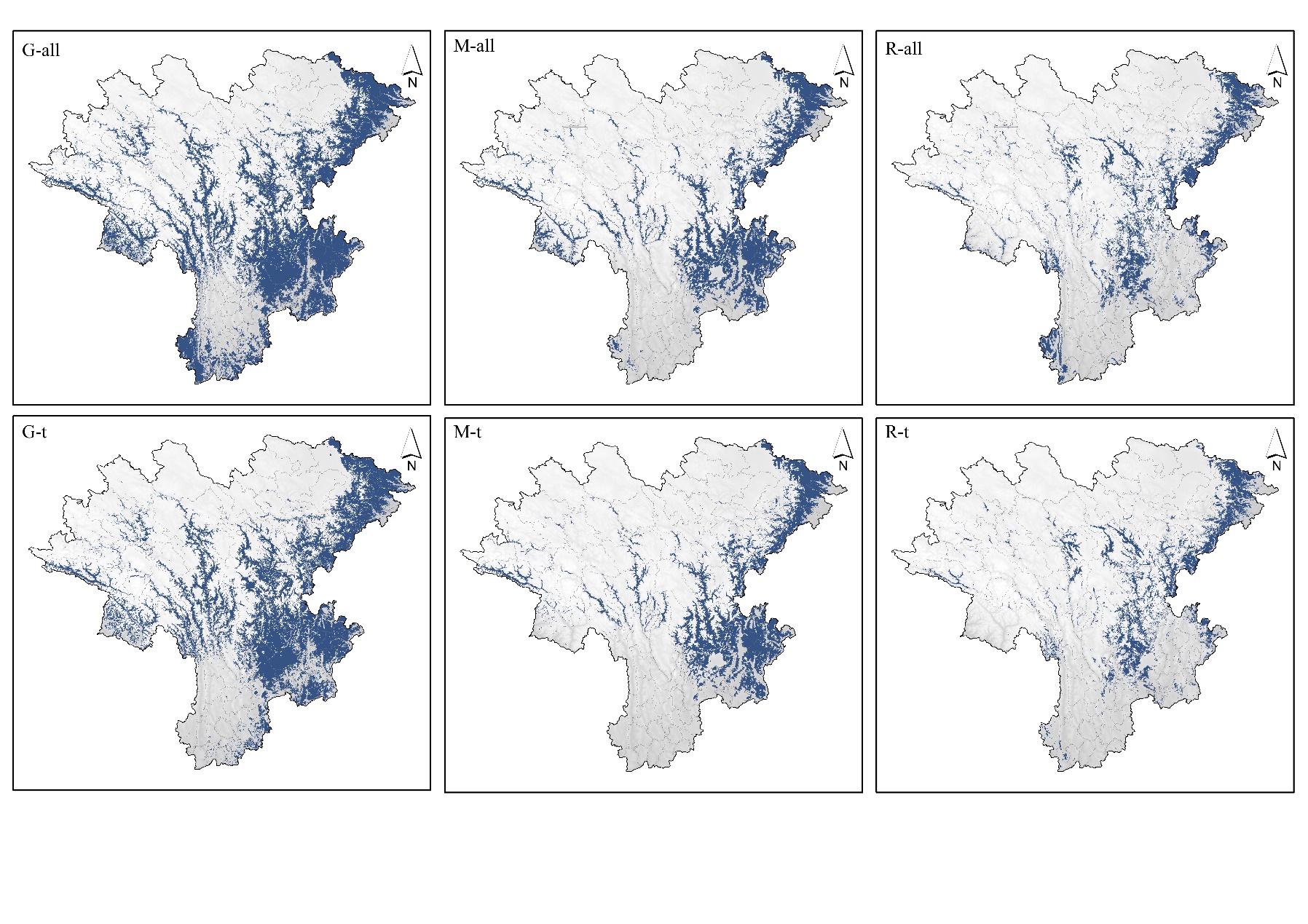


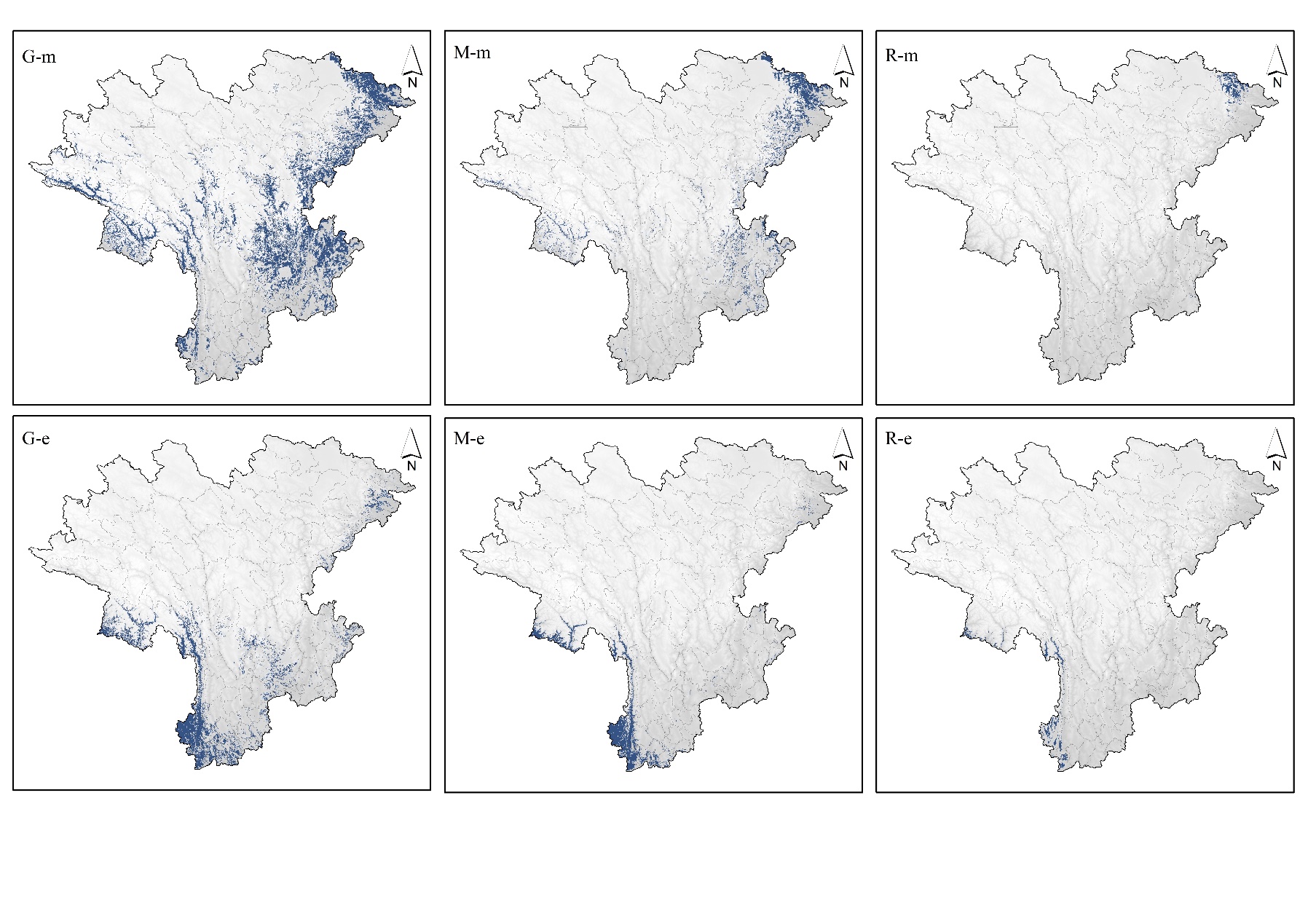


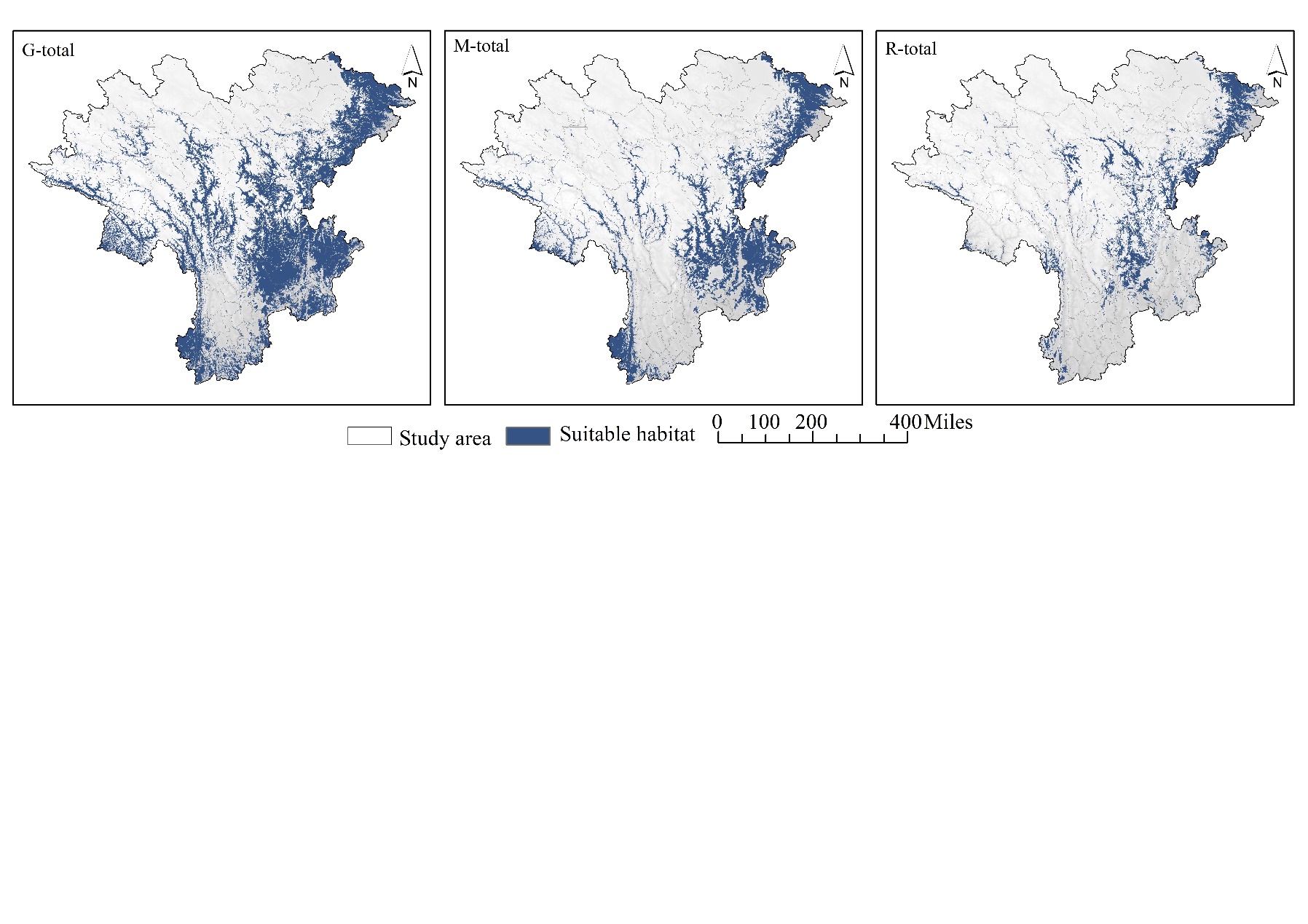

Supplement: Supplementary file 2 — Appendix S1–S2 [file ECE3-13-e10566-s002.docx]
